# Moyamoya Disease with Peripheral Pulmonary Artery Stenoses and Coronary Artery Fistulae

**DOI:** 10.1155/2009/840904

**Published:** 2009-05-27

**Authors:** Lindsay Reardon, Andrew O. Maree, Michael de Moor

**Affiliations:** ^1^University of Colorado School of Medicine, Aurora, CO 80045, USA; ^2^Department of Medicine, Boston Medical Center and Massachusetts General Hospital, Boston, MA 02114, USA; ^3^Pediatric Cardiology, MassGeneral Hospital for Children and Harvard Medical School, CRP-S 510, Boston, MA 02114, USA

## Abstract

Moyamoya is a progressive disorder of the cerebral vasculature. Our report describes a rare case of Moyamoya disease with distal peripheral pulmonary artery stenoses and coronary fistulae in a 12-year-old Caucasian female patient.

A 12-year-old Caucasian female with known Moyamoya was admitted for hemodynamic and angiographic evaluation of increasing dyspnea and exertional chest pain. Echocardiogram indicated elevated right ventricular pressure. The patient had initially presented one year previously with acute onset of neurological symptoms, which included dysarthria and involuntary movements. Her developmental history to that point had been entirely normal. A Computed Tomography angiogram demonstrated severe narrowing of the cervical and intracranial carotid arteries bilaterally, consistent with an advanced Moyamoya pattern. Pial synangiosis was performed with a subsequent improvement in her symptoms. 

 At cardiac catheterization she was shown to have an elevated pulmonary artery pressure (75/18 mmHg, mean 36). Angiography showed that the right coronary artery was patent, and there were two fistulae (Figure 1). The fistulae (indicated by arrows) appear to connect the proximal right coronary artery to the right atrium and the mid vessel to the small cardiac vein. Pulmonary angiography revealed extensive distal peripheral arterial stenoses and areas of pulmonary hypovascularity (Figure 2). Left and right magnetic resonance carotid and cerebral angiography demonstrated marked attenuation of the right internal carotid artery and occlusion of the left internal carotid artery (Figure 3).

Moyamoya is a rare progressive disorder of the cerebral vasculature characterized by bilateral carotid artery occlusive disease that was first described in 1957 (Figure 3) [1]. The resulting dense abnormal collateral vascular network at the base of the brain has the angiographic appearance of a “puff of smoke” or, in Japanese, “moyamoya.” Arterial involvement of the renovasculature occurs in 5% of cases [2]. There are limited reports of involvement of the coronary and pulmonary circulation. Epicardial coronary artery stenoses may occur, and one case describes right coronary artery to right ventricular fistulae in a 56-year-old male [3]. Another one reports systemic and pulmonary hypertension in a child with Moyamoya disease [4]. Our case is a rare description of Moyamoya disease with distal peripheral pulmonary artery stenoses and coronary fistulae in a young female patient.

## Figures and Tables

**Figure 1:**
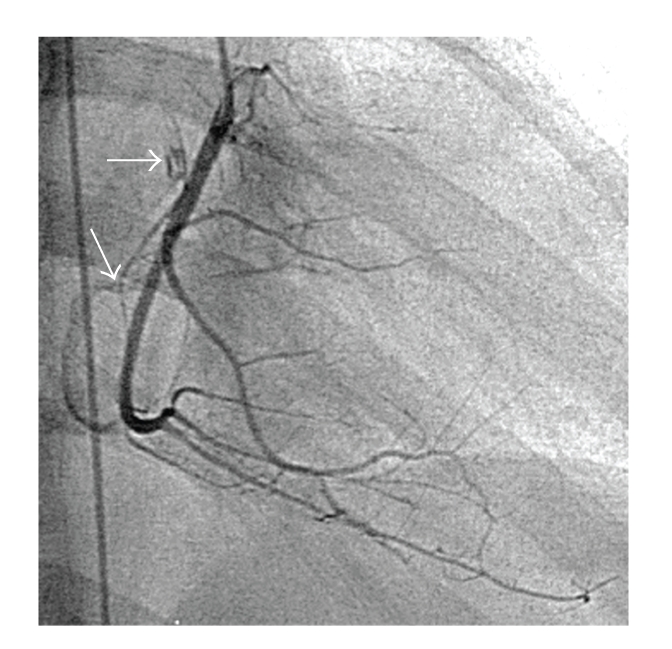
Right coronary angiogram in a straight right anterior-oblique projection demonstrating two fistulae (indicated by arrows). The first fistula connects the proximal right coronary artery to the right atrium, and the second appears to connect the mid-vessel to the small cardiac vein.

**Figure 2:**
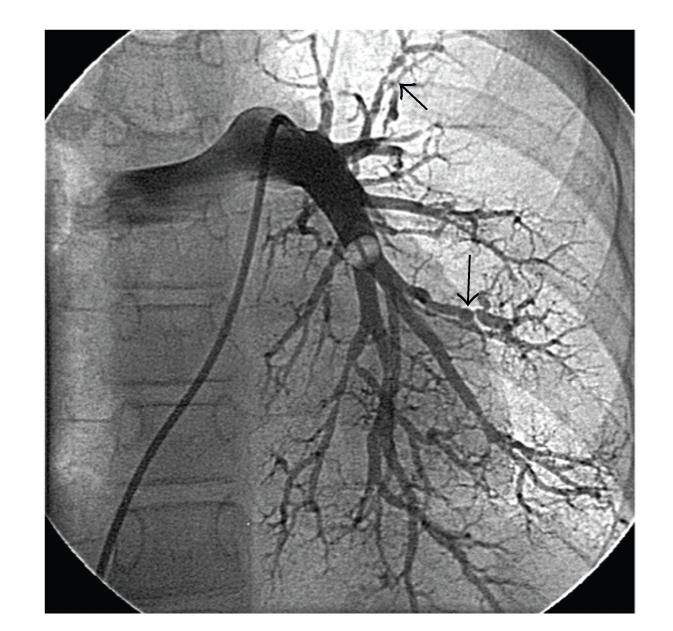
Left main pulmonary angiogram demonstrating extensive distal peripheral arterial stenoses (indicated by arrows) and areas of pulmonary hypovascularity.

**Figure 3:**
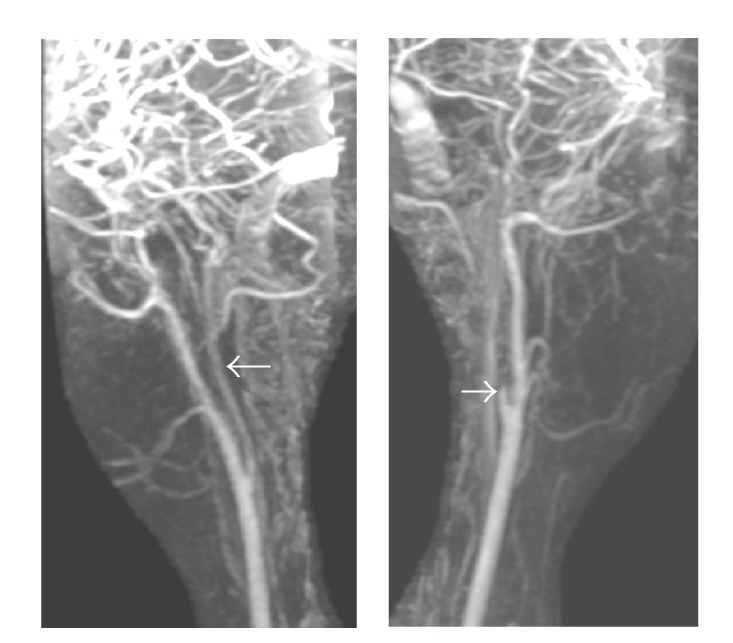
Composite image of left and right magnetic resonance carotids and cerebral angiographies. Left panel demonstrates marked attenuation of the right internal carotid artery (indicated by arrow) and right panel shows occlusion of the left internal carotid artery (indicated by arrow).
